# Recent patterns in chronic disease mortality in remote living Indigenous Australians

**DOI:** 10.1186/1471-2458-10-483

**Published:** 2010-08-16

**Authors:** K Andreasyan, WE Hoy

**Affiliations:** 1 Centre for Chronic Disease, The University of Queensland, Royal Brisbane and Women's Hospital, Brisbane, Queensland, Australia

## Abstract

**Background:**

Despite the well-recognised Indigenous-non-Indigenous health disparity, some reports suggest improvements in Indigenous mortality. Our aim was to quantify Indigenous mortality in Outer Regional (OR), Remote (R), and Very Remote (VR) areas in New South Wales, Queensland, South Australia, Western Australia, and the Northern Territory and changes in mortality from 1998 to 2005.

**Methods:**

We calculated rates, standardized mortality ratios (SMRs) and percentage change in annual rates of Indigenous cardiovascular, diabetes and renal mortality mentioned anywhere on the death certificate by using ICD-10 codes and the 2001 total Australian population as the reference population.

**Results:**

In 1998-2001, Indigenous SMRs for all-cause mortality were 241%, 421% and 220% in OR, R and VR, respectively. In 2001-03, corresponding SMRs were 202%, 331% and 176%. Percentage changes (95% confidence interval) in annual all-cause mortality were -3.0% (-5.3%, -0.7%) in OR, -4.2% (-7.4%, -0.9%) in R and -0.5 (-9.1%, -0.7%) in VR. In 2002-2005, compared with 1998-2001, changes in the number of Indigenous deaths were -147, -195, and -197 in OR, R and VR, respectively. Similar patterns and trends were observed for cardiovascular mortality.

**Conclusions:**

Mortality was elevated about 2-fold in OR, 4-fold in R and 2-fold in VR areas. The downward trend in mortality regardless of remoteness of residence was partly attributable to a decrease in the absolute number of deaths. These patterns were observed for each of the states/territories individually.

## Background

Indigenous Australians have a life expectancy about 17 years less on average than the general Australian population [1] and experience much higher mortality rates [2-5]. Repeated reports on the Indigenous-non-Indigenous health disparity have contributed to substantial criticism of health services, and pessimism about the potential for improvement in Indigenous mortality [6,7]. However, reports from the Northern Territory (NT) [8-10] and Western Australia [11] suggest that Indigenous mortality rates are improving. A recent Australian Institute of Health and Welfare study reported decline in all-cause death rates in all areas, particularly in remote areas, where rates tend to be higher [12]. These trends were largely attributed to improvements in Indigenous mortality.

Methodological differences between the available studies hinder their comparison. Up until 1996, only underlying causes of death were available; this yielded somewhat insensitive data, which, among other limitations, greatly underestimate the association of diabetes and particularly renal disease with deaths [13]. In addition, there are gradations of mortality rates by region, with remote communities said to have the highest rates [12]. Thus mortality analyses should be based on multiple causes of death and take remoteness of residence into account.

In two recent reports, analyzing multiple causes of death, we showed that Indigenous mortality in the NT [15] and in Queensland (unpublished study) was highest in people in remote rather than in very remote areas. Furthermore the data showed a downward trend in mortality from 1997 to 2004. There were improvements in all-cause, cardiovascular, diabetes and renal mortality, which were most pronounced in remote areas in the NT and in very remote areas in Queensland and which were partly attributable to a decrease in the absolute number of deaths. A similar remoteness gradient with the highest mortality in remote rather than very remote areas was reported in a study from South Australia [15].

Our aim in the current study was to obtain recently available data and examine nationwide patterns in Indigenous chronic disease mortality from 1998 to 2005 by multiple causes of death and remoteness of residence to see if the patterns observed in Queensland and the NT applied more broadly.

## Methods

The Australian Bureau of Statistics (ABS) provided de-identified mortality data for all deaths in Australia from 1998 to 2006 and estimates of the Indigenous and Torres Strait Islander [16] and total resident [17] populations. Although Indigenous status has been included in death notification forms in places like the NT since 1988 [1], multiple cause of death data have been compiled for Australia only since 1997 [18]. Since 1997 there have also been substantial improvements in the quality of Indigenous mortality data [18].

Victoria, Tasmania and the Australian Capital Territory were excluded from this analysis because of small numbers of Indigenous deaths, small population numbers and very poor mortality coverage [1], i.e. Indigenous status ascertainment in data. Data from New South Wales, Queensland, South Australia, Western Australia, and the NT were included, where coverage of Indigenous deaths ranged from 45% in New South Wales to 94% in the NT during the study period [1]. Because coverage is believed to be more complete in more remote areas [12], our analyses were restricted to Outer Regional, Remote and Very Remote areas in these states and territories. Furthermore, we conducted the analyses with and without the NSW data, which has the poorest Indigenous death coverage and identification among the analysed jurisdictions.

Causes of deaths were defined in accordance with the International Classification of Diseases, 10^th ^revision, as follows: cardiovascular - I10-11, I14-15, I20-25, I42, I44-52, I60-74, diabetes - E10, 11, 13, 14, renal - E10.2, E11.2, E12.2, E13.2, E14.2, I12, I13, I15.0, I15.1, I70.1, N00-N29, N39, R80-82. In the analyses, the term 'multiple cause' or 'cause mention' was referred to cause of death mentioned anywhere on the death certificate [18].

We defined the categories of remoteness by the Accessibility and Remoteness Index of Australia (ARIA) which is based on the distance people must travel along a road network to get to a service centre. The index is more completely described elsewhere [19]. The analysed categories of remoteness (ARIA score range) included Outer Regional (2.4 to <5.95), Remote (5.95 to <10.5), and Very Remote (≥10.5).

The analyses were restricted to people aged 25 and over because the events of interest were uncommon in younger people. Average annual crude death rates for the 1998-2001 and 2002-2005 periods were age-adjusted to the 2001 total Australian population by using 25-44, 45-64 and 65+ yr age groups. Because of overdispersion of death data, the average annual percentage change in death rates over the entire eight year period was estimated by negative binomial rather than by Poisson regression. The difference in age structures between the Indigenous and nationwide populations was accounted for by calculating standardised mortality ratios (SMRs), through indirectly standardising age-, sex-, period- and region-specific Indigenous rates to the 2001 total Australian population (reference population). SMRs were calculated by dividing the number of deaths actually observed by the number of deaths expected if the Indigenous population had the same age and sex distribution as the reference population. As a ratio of observed to expected deaths, SMR >100% means that there were more deaths than expected. P < 0.05 was considered statistically significant. The analyses were conducted by using STATA 10.

## Results

Table 1 shows age-adjusted rates of all-cause, cardiovascular, diabetes and renal Indigenous mortality by remoteness of residence. Indigenous people in Remote areas experienced higher all-cause, cardiovascular, diabetes and renal mortality rates in both study periods compared to their Indigenous counterparts in Outer Regional and Very Remote areas. For example, in 1998-2001, all-cause death rates (per 1,000) [95% confidence interval (CI)] were 28.6 (26.1, 31.1), 47.4 (42.8, 52.0) and 24.7 (22.4, 27.0) in Outer Regional, Remote and Very Remote areas, respectively. In 2002-2005, the corresponding rates (95% CI) were 24.5 (22.2, 26.8), 35.7 (31.8, 39.6) and 19.9 (18.0, 21.8). The Table also shows that the rates declined most in Remote areas. Similar patterns were observed for cardiovascular, diabetes and renal mortality.

**Table 1:** Average annual age-standardised rates per 1,000 population (95% confidence interval) of all-cause and cause-specific Indigenous mortality.

	Outer Regional		Remote		Very Remote	
Cause of death	1998-2001	2002-2005	1998-2001	2002-2005	1998-2001	2002-2005
All cause	28.6 (26.1 - 31.1)	24.5 (22.2 - 26.8)	47.4 (42.8 - 52.0)	35.7 (31.8 - 39.6)	24.7 (22.4 - 27.0	19.9 (18.0 - 21.8)
Cardiovascular	17.3 (15.2 - 19.4)	14.5 (12.6 - 16.4)	26.5 (22.9 - 30.1)	19.1 (16.1 - 22.1)	12.8 (11.2 - 14.5)	10.7 (9.2 - 12.2)
Diabetes	7.1 (5.7 - 8.5)	6.4 (5.1 - 7.7)	8.8 (6.7 - 10.9)	7.7 (5.8 - 9.6)	5.3 (4.2 - 6.4)	4.6 (3.6 - 5.6)
Renal	5.1 (3.9 - 6.3)	4.6 (3.5 - 5.7)	9.5 (7.2 - 11.8)	7.9 (6.0 - 9.8)	5.6 (4.5 - 6.7)	4.8 (3.8 - 5.8)

See **Methods **for definitions of causes of deaths and remoteness categories

**Figure 1:**
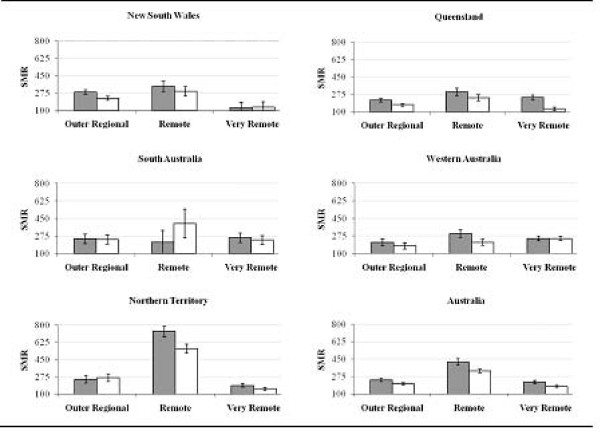
**Standardised mortality ratios (95% confidence interval) of all-cause Indigenous mortality**. SMR: standardised mortality ratio. Grey square: 1998-2001, white square: 2002-2005. See **Methods **for definitions of causes of deaths and remoteness categories. Reference population for calculating standardised mortality ratios: 2001 total Australian population. A standardised mortality ratio equal to 100% means that, compared to the total population, there are no more Indigenous deaths than expected.

Table 2 shows that the disparity between Indigenous and nationwide all-cause and cause-specific mortality was also largest in Remote areas, where SMRs (95% CI) for all-cause mortality were 421% (399%, 443%) in 1998-2001 and 331% (313%, 349%) in 2002-05. In Outer Regional areas the corresponding SMRs were 241% (230%, 252%) and 202% (191%, 213%), and in Very Remote areas - 220% (209%, 231%) and 176% (167%, 185%). Similar patterns were observed for cardiovascular, diabetes and renal mortality. When we analysed the all-cause mortality data by state/territory (Figure 1), the NT had the highest Indigenous mortality. Remote areas showed the largest Indigenous-non-Indigenous disparity and the greatest improvements in disparity over the study period. The only exception was South Australia, where the disparity increased in Remote areas. However, because of small numbers, the latter observation must be interpreted with caution.

**Table 2:** Standardised mortality ratios, % (95% confidence interval), of all-cause and cause-specific Indigenous mortality.

	Outer Regional		Remote		Very Remote	
Cause of death	1998-2001	2002-2005	1998-2001	2002-2005	1998-2001	2002-2005
All cause	241 (230 - 252)	202 (191 - 213)	421 (399 - 443)	331 (313 - 349)	220 (209 - 231)	176 (167 - 185)
Cardiovascular	269 (252 - 286)	221 (206 - 236)	427 (396 - 458)	328 (302 - 354)	204 (190 - 218)	170 (157 - 183)
Diabetes	636 (573 - 699)	554 (497 - 611)	826 (721 - 931)	727 (633 - 821)	513 (458 - 568)	416 (369 - 463)
Renal	401 (356 - 446)	351 (310 - 392)	742 (652 - 832)	631 (551 - 711)	428 (382 - 474)	363 (323 - 403)

See **Methods **for definitions of causes of deaths and remoteness categories

Reference population for calculating standardised mortality ratios: 2001 total Australian population

Standardised mortality ratio: number of observed deaths/number of expected deaths. Expected deaths calculated by applying age-, sex-, period- and region-specific Indigenous death rates to the reference population. A ratio equal to 100% means that, compared to the total population, there are no more Indigenous deaths than expected.

The average annual percentage changes (95% CI) in all-cause death rates over the entire eight year period were significant in all remoteness areas and were -3.0% (-5.3%, -0.7%), -4.2% (-7.4%, -0.9%) and -0.5 (-9.1%, -0.7%) in Outer Regional, Remote and Very Remote areas, respectively. Cardiovascular mortality also significantly declined in all the areas, with corresponding annual percentage changes (95% CI) of -4.6% (-7.2%, -1.8%), -6.5% (-8.7%, -4.2%) and -5.4% (-7.5%, -3.2%). Significant declines were also observed for diabetes deaths in Outer Regional areas (-3.5% (-6.8%, -0.4%)) and Very Remote areas (-4.2% (-7.5%, -0.8%)) and for renal deaths in Remote areas (-4.7% (-8.3%, -0.8%). The observed declines in mortality were due not only to an increase in numbers of the Indigenous population, but also to a decrease in the numbers of actual deaths (Table 3). Whereas the number of all-cause deaths over the study period decreased in Remote areas in all states and territories, the main drop in numbers of deaths in Very Remote areas was attributable to those trends in Queensland and the NT (Table 4).

**Table 3:** Numbers of cause-specific Indigenous deaths, Australia*.

	Outer Regional		Remote		Very Remote	
	1998-2001	2002-2005	1998-2001	2002-2005	1998-2001	2002-2005
All-cause deaths	1664	1517	1361	1166	1587	1390
Cardiovascular deaths	928	827	696	577	760	685
Diabetes deaths	369	349	223	218	313	279
Renal deaths	281	267	245	227	321	298
Mid-period population	34,708	38,109	15,778	17,369	32,667	35,957

* includes New South Wales, Queensland, South Australia, Western Australia, and the Northern Territory.

See **Methods **for definitions of causes of deaths and remoteness categories.

The numbers of cause-specific are multiple cause of deaths numbers and do not sum up to the number of all-cause deaths.

**Table 4:** Numbers of all-cause Indigenous deaths by state/territory.

	Outer Regional		Remote		Very Remote	
	1998-2001	2002-2005	1998-2001	2002-2005	1998-2001	2002-2005
New South Wales	555	473	168	155	26	27
Queensland	667	572	270	235	427	245
South Australia	106	115	32	15	116	118
Western Australia	149	132	268	200	466	490
Northern Territory	187	225	640	544	552	510

See **Methods **for definitions of causes of deaths and remoteness categories

## Discussion

In both study intervals, the Indigenous population in Australia had higher rates of all-cause, cardiovascular, diabetes and renal mortality compared to the general Australian population. However, Indigenous people in Remote areas showed higher all-cause, cardiovascular, diabetes and renal mortality rates than Indigenous people in Outer Regional and Very Remote areas.

There was a downward trend in rates and SMRs of Indigenous mortality in all states/territories over the two consecutive time periods. The trend was significant for all-cause and cardiovascular mortality in all remoteness areas, for diabetes mortality - in Outer Regional and Very Remote areas and for renal mortality - in Remote areas. These downward changes were due not only to an increase in the Indigenous population, but also, more importantly, to decrease in the number of deaths over time. The differentials by areas of remoteness and the decline in rates over time were observed for each of the states/territories individually.

This study shows show that previously described findings of the lowest mortality rates in Very Remote areas in the NT [14] and Queensland (unpublished study) are also seen in other states with reasonable coverage of Indigenous deaths. Furthermore it supports the observation of the trend towards lower rates in recent years.

For deaths from diabetes and renal causes, which are less likely to be reported as the underlying cause of death [13], analyses by multiple causes more completely describes the mortality. This study has some potential limitations. Indigenous misclassification is a large problem in urban areas. Limiting analyses to remote communities, where ascertainment is considered to be good and there is believed to have been little change in the 'propensity to self-identify' as Indigenous [19], minimised the impact of the misclassification. Yet, even in the study regions, Indigenous ascertainment is not perfect and the results should be interpreted with caution. Availability of data up to 2006 allowed investigation of recent mortality patterns and minimised the effect of late registration of deaths on the results by including deaths which occurred in 2005 but were registered in 2006. Studies of longer duration will allow investigation of more long-term trends in Indigenous mortality. When New South Wales, which has the worst coverage of Indigenous deaths among the study areas, was excluded from the analyses, the results were little changed (data not shown).

The ARIA classification of remoteness is simple and sound for statistical purposes [19]. However, the concept is subject to criticism because the index relies on road distance as a surrogate for remoteness and on the population size as a surrogate for the availability of services; the index does not look at which services are available in a given town [19]. For example, in the NT, the suburbs of Darwin are classified as Outer Regional, Alice Springs, Jabiru and Katherine are classified as Remote, and Bathurst and Melville islands, Tennant Creek, East and West Arnhem, and the Gulf communities are classified as Very Remote. The complete list of statistical local areas by remoteness categories in the NT and other study areas can be found elsewhere [20].

The higher rates of all-cause and the cause-specific mortality rates in Remote areas compared to Outer Regional and Very Remote areas are contrary to most current dogma, which specifies that rates are highest in Very Remote areas. The reasons for our findings are speculative. Selection bias is likely to be operating in that people from Very Remote areas move to population centres for better access to health services for themselves or sick family members. Preston-Thomas *et al *found that 78% of Indigenous people with end stage renal disease living remotely had to relocate to access renal replacement therapy [21]. When these patients ultimately succumb, they may contribute to the death toll in the area of treatment. In addition, the death rates may reflect the greater availability of alcohol and potentially other drugs in the population centres. The contribution of these phenomena is, however, uncertain. Better understanding of migration and length of usual residence in a particular setting, and the distinctions between area of origin and area of recent residence as captured both by the census and death certificates, are necessary for accurate interpretation of the findings.

It is also possible that Indigenous health is indeed better in Very Remote than Remote areas due to a more favourable social environment [22], better family support, increased physical activity [23], a healthier diet [24], and lower rates of substance [25] and alcohol abuse [22]. A recent study by Burgess *et al *[26] showed that caring for country was associated with significantly more frequent physical activity, better diet, lower body mass index, less abdominal obesity, lower systolic blood pressure, less diabetes, non-elevated albumin-creatinine ratio, higher high density cholesterol lipoprotein cholesterol level, lower cardiovascular risk and reduced mortality in Indigenous Australians. In a recent study by Rowley *et al *[27], lower than expected cardiovascular morbidity and all-cause mortality in a decentralized Aboriginal community in the Northern Territory were attributed to regular primary health care services, better physical activity and diet, limited access to alcohol, social factors and self-determination.

The reasons for improvements in chronic disease mortality are also largely speculative. Chronic diseases such as cardiovascular disease, diabetes and renal disease are associated with factors such as a sedentary lifestyle, poor nutrition [28], excessive alcohol consumption [29], low birthweight, infections, cigarette smoking, and poverty and educational disadvantage. Changes in these factors might partly explain changes in chronic disease mortality. Improved birthweights between the 1960 s and 1980 s [30,31] and reduced infections [32,33] are among possible ameliorating factors. Health promotion programs targeting nutrition, exercise, smoking and alcohol [34,36] might also play a role. In addition, management of chronic disease has undoubtedly improved in most Indigenous health care settings across Australia since the mid 1990 s. Approaches include screening asymptomatic people at intervals for chronic disease and treatment of people with high blood pressure, renal disease, high glucose levels and disordered lipid levels [37,38]. Although these approaches have not been as systematic or as adequately resourced as one would hope, they should, if sustained, ultimately be reflected in better outcomes, including mortality rates.

## Conclusions

This study suggests that the reduction in death rates of Indigenous people observed in the NT and Queensland applies nationally and provides some grounds for optimism. Future studies with a longer observational period, a focus on migration and a better classification of remoteness are required to understand and support the contributing factors and strategies. Our future work will investigate longer-term changes in Indigenous mortality, the use of antihypertensive, renal protective, lipid lowering and hypoglycemic medicines in remote-living Indigenous people and its potential effects on trends in mortality. Identified gaps can support arguments for more resources, needs-based resource allocation and ongoing modification of health policy in remote and very remote areas taking migration into account. Assessment of various health service models will also form part of future research.

## Competing interests

The authors declare that they have no competing interests.

## Authors' contributions

KA acquired, analysed and interpreted the data and drafted the manuscript. WEH contributed to conception and design of the study and revision of the manuscript. All authors read and approved the final manuscript.

## Pre-publication history

The pre-publication history for this paper can be accessed here:

http://www.biomedcentral.com/1471-2458/10/483/prepub
